# Role of Redox Status in Development of Glioblastoma

**DOI:** 10.3389/fimmu.2016.00156

**Published:** 2016-04-26

**Authors:** Aleli Salazar-Ramiro, Daniela Ramírez-Ortega, Verónica Pérez de la Cruz, Norma Y. Hérnandez-Pedro, Dinora Fabiola González-Esquivel, Julio Sotelo, Benjamín Pineda

**Affiliations:** ^1^Neuroimmunology and Neuro-Oncology Unit, National Neurology and Neurosurgery Institute (INNN), Mexico City, Mexico; ^2^Neurochemistry Unit, National Neurology and Neurosurgery Institute (INNN), Mexico City, Mexico; ^3^Experimental Oncology Laboratory, National Cancer Institute, Mexico City, Mexico

**Keywords:** glioblastoma, gliomagenesis, redox status, DNA damage, tumor microenvironment

## Abstract

Glioblastoma multiforme (GBM) is a highly aggressive neoplasia, prognosis remains dismal, and current therapy is mostly palliative. There are no known risk factors associated with gliomagenesis; however, it is well established that chronic inflammation in brain tissue induces oxidative stress in astrocytes and microglia. High quantities of reactive species of oxygen into the cells can react with several macromolecules, including chromosomal and mitochondrial DNA, leading to damage and malfunction of DNA repair enzymes. These changes bring genetic instability and abnormal metabolic processes, favoring oxidative environment and increase rate of cell proliferation. In GBM, a high metabolic rate and increased basal levels of reactive oxygen species play an important role as chemical mediators in the regulation of signal transduction, protecting malignant cells from apoptosis, thus creating an immunosuppressive environment. New redox therapeutics could reduce oxidative stress preventing cellular damage and high mutation rate accompanied by chromosomal instability, reducing the immunosuppressive environment. In addition, therapies directed to modulate redox rate reduce resistance and moderate the high rate of cell proliferation, favoring apoptosis of tumoral cells. This review describes the redox status in GBM, and how this imbalance could promote gliomagenesis through genomic and mitochondrial DNA damage, inducing the pro-oxidant and proinflammatory environment involved in tumor cell proliferation, resistance, and immune escape. In addition, some therapeutic agents that modulate redox status and might be advantageous in therapy against GBM are described.

## Introduction

Central nervous system (CNS) tumors are the most common neoplasia in pediatric patients under 19 years old. In adults, glioblastoma multiforme (GBM) is the most common aggressive tumor of the CNS. In Mexico, GBM represents 28% of all gliomas and 9% of all neoplasms (1). High intra- and intertumor heterogeneity, diffuse brain infiltration, necrosis, high rate of cell proliferation, and resistance to current treatments characterize these tumors (2, 3).

Glioblastoma multiforme has two origins: tumors arising *de novo*, called primary GBM that represent 90–95% of all GBMs; they are usually diagnosed between the sixth and seventh decades of life. The remaining 5–10% of them (named secondary) arise from lower grade tumors through several genetic mutations, such as retinoblastoma protein (RB), phosphatase and tensin homolog (PTEN), and vascular endothelial growth factor receptor (VEGFR), and other mutations, which finally lead to p53 inhibition, overexpression of platelet-derived growth factor A receptor-α (PDGFA/PDGFRα) and amplified cyclin-dependent kinase 4 (CDK4) (4, 5). Secondary GBM is commonly diagnosed around the fourth decade of life. Despite multiple advances in diagnosis and treatment, prognosis for GBM is poor; survival for untreated tumors is about 5 months. Even the best available current therapy (which includes surgery, chemotherapy, and radiotherapy) works only as a palliative and median survival does not extend beyond 14 months (6).

Although several reports have established the existence of cancer stem cells-like populations within the GBM and several experimental models have demonstrated that transformed neural stem/precursor cells are probably the origin cell of those tumors, conclusive evidence remains missing (3, 7–9).

The genesis, development, and progression of GBM and its resistance to standard treatments remains obscure; however, it is thought that GBM cell mechanisms involve clonal and sub-clonal populations from both the subventricular zone (SVZ) cell population that includes carcinogenic stem cells (CSC) and a mixture of tumor mass population, which in turn includes astrocytes, microglia, non-differentiated cells, and partially differentiated cells (10). Some authors have agreed that it involves a multistep process including a series of mutations and activation of several oncogenes. These cell populations might suffer genetic alterations caused by different factors such as ultraviolet and ionizing radiation (IR), carcinogens, and oxidative stress (11).

## Gliomagenesis

Gliomagenesis is a multistep process where genetic alterations on normal cells may lead to malignant derivatives (secondary glioblastoma) or to highly malignant transformed cells (primary glioblastoma) (4) when multiple mutations are involved. There are several hypotheses about the onset of gliomas and their progression through glioblastoma. Histopathologic features of primary and secondary GBMs are indistinguishable; nevertheless, molecular genetic abnormalities are associated with each subtype.

Primary GBMs exhibit epidermal growth factor receptor (EGFR) amplification, PTEN mutation, and loss of chromosome 10, while P53 mutations are common in secondary GBMs (12). These mutations affect the redox balance in the tumor environment. For instance, ligation of EGFR by EGF induces endogenous production of intracellular reactive oxygen species (ROS) and H_2_O_2_ in cancer cell lines (13, 14). In response to ligation, EGFR forms homo and heterodimers activating several intracellular signal pathways, such as phosphatidylinositol 3′ kinase (PI3K)/Akt and Ras/mitogen-activated protein kinase (MAPK), leading to increase in DNA synthesis (13). Also high levels of H_2_O_2_ (200 pM) significantly increase the Tyr autophosphorylation by EGFR, leading to generation of ROS (13).

Phosphatase and tensin homolog is known by acting as a tumor suppressor, negatively regulating PI3K/Akt pathway (15, 16). This protein plays an important role in the regulation of metabolism, apoptosis, cell proliferation, and survival, being affected by redox status, specifically by H_2_O_2_, which can oxidize the protein, inducing the formation of a disulfide bond between Cys71 and Cys124 in the N-terminal phosphatase domain (17). As a result, this leads to alterations in its interaction with signaling and regulatory proteins (17–19). Then, it is possible that overexpression of EGFR might conduce to an increase in H_2_O_2_ levels, disturbing several signaling pathways and stimulating cell survival and proliferation.

Tumor protein P53 (P53) is a protein that regulates the energetic metabolism and the genes involved in the redox regulation, such as mitochondrial superoxide dismutase 2 (SOD2) (20), glutathione peroxidase 1 (GPX1) (21), and the aldehyde dehydrogenase 4 family member A1 (ALDH4A1) (22). P53 may be affected by several mutations that change its structure and function. Patients with Germline mutations in *TP53* and pR337H show higher levels of oxidant stress (23).

This genetic heterogeneity separates GBM subtypes and is defined by gene expression analysis. Novel therapeutic alternatives are now focused to increase the immune recognition and immune response (24, 25), to block metabolism pathways (26), to knock genes (27), and to modulate cellular redox status (28).

Chronic inflammation in various tissues is a critical component of tumor development (29). In the case of brain tumor malignancy, no conclusive links have been found between glioma and smoking, diet, mobile phones, or electromagnetic fields. Only IR has been accepted as the risk factor (30) due to its ability to induce DNA damage response and repair (DDR/R) (31). When the cell is damaged by IR, it can inherit to its offspring several mutations or enter to apoptosis or to a senescence status (31). Apoptotic bodies and senescence cells are phagocyted by the mononuclear phagocyte system (32). One of the main effectors of the DDR/R pathway is P53 that also plays a key role in the induction of the proinflammatory response (33). DNA damage induced by radiation allows the release of damage-associated molecular pattern (DAMP) (34). Also, some viral infections, such as JC virus, BK virus, simian virus 40 (35), cytomegalovirus (CMV), and Measles virus (recently postulated) (36, 37), have been implicated in the genesis of brain tumors. Therefore, it is postulated that some tumors may arise from tissues that were damaged by infections or chronic inflammation (38). Virus have the ability to cause lytic infection in permissive cells and to remain in latency in other cell types, such as astrocytes, neurons, myeloid progenitor cells, and/or lymphocytes. Besides, they are candidates to produce persistent cell infection, activating, and modulating immune response, either through Toll-like receptors (TLRs) or by additional mechanisms of activation of TLRs, inducing endogenous inflammatory DAMPs mediators that also participate in the immune response (39) against pathogen-associated molecular patterns (PAMPs). DAMPs are nuclear and cytosolic proteins, nucleotides, and extracellular molecules (40). TLR’s activation by PAMPs and DAMPs causes overexpression of pro-inflammatory cytokines and costimulatory molecules involved in the generation of the immune response (41). Infections can also activate the DDR/R pathway and induce the release of IFNα/β, activating p53 and inducing apoptosis, which is relevant for an adequate antiviral immune response and tumor suppression (42). Also, DAMPs and PAMPs activate NFκB, PI3K/AKT, and Ras/MAPK signaling, favoring cell proliferation (43), allowing TNF-α and IL-6 release and perpetuating tissue damage due to inflammation (44). The activation of DDR/R as response to viral infection is ROS dependent (45, 46). All these processes lead to changes in the interstitial microenvironment as a result of infections or sustained inflammation; thus, it seems possible that they can drive to tumor initiation and progression *via* the release of ROS by activated immune cells (40).

**Figure 1:**
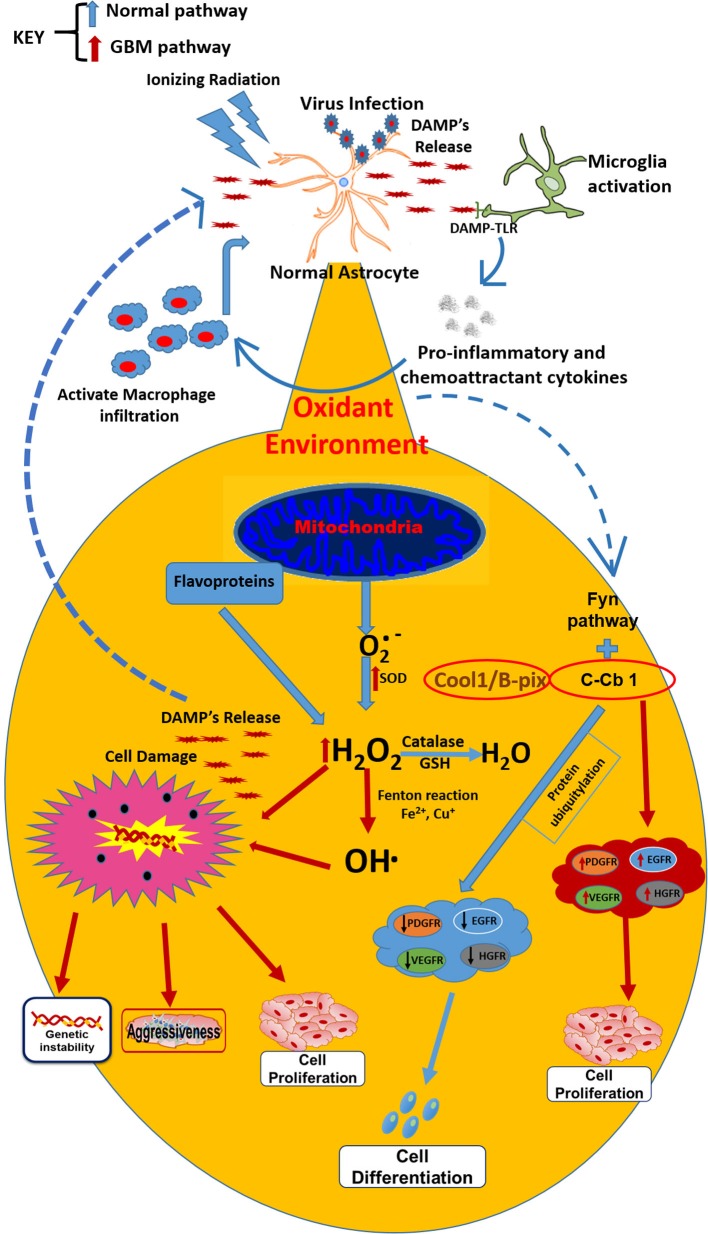
**Under normal conditions, the** ${\text{O}}_{2}^{\;•-}$ **secreted by mitochondria is transformed by catalase action and GSH into H_2_O**. However, when external factors (ionizing radiation or virus) initiate the astrocytes transformation into astrocytoma cells, microglia are activated conducting to the release of proinflammatory cytokines, macrophages infiltration, and DAMP’s release, which are recognized by infiltrated macrophages and TLRs on the surface of microglia (resident macrophages). These changes lead to an oxidant environment where the ${\text{O}}_{2}^{\;•-}$ is transformed by SOD (which is overexpressed in tumor cells) into H_2_O_2_ and OH^⋅^, which drives to metabolic changes and chromosomal instability and finally, leading to resistance, aggressiveness, and cell proliferation. Besides, the oxidant environment activates the FyN pathway, which under normal conditions, with the activation of Fyn kinase plus C-Cb1, produces ubiquitination of growth factor receptors and posterior degradation, directing to cell differentiation. In GBM cells, the sequestration of C-Cb1 by Cool-1/B-Pix avoids the degradation of such receptors, increasing their expression and guiding to cell proliferation in GBM environment.

It is difficult to know which event is the first to trigger gliomagenesis, whether there is a DNA alteration as result of an imbalance in the redox homeostasis or if the imbalance in the redox state involves alterations in key genes that promote gliomagenesis. However, chronic inflammatory process could also result in the development of GBM (47), Moreover, it is recognized that inflammation is linked to redox modulation; tumor cells are under pro-oxidant redox environment due to an increased production of ROS (48). TNF-α is a cytokine released during the inflammatory processes, induced by microorganisms or IR and is the prime mediator of inflammation; its signaling can activate signaling pathways pro- and anti-apoptotic and is elevated in GBM. In glioblastoma cells, TNF-α increases the ROS production (49). Among the signaling pathways that are activated by TNF-α, is the PI3K/Akt (involved in regulating cell growth and apoptosis resistance), but is unregulated in GBM (50), leading to cell proliferation and survival. Akt phosphorylation is redox state-dependent (51) and has been shown that GBM human cells exposed to TNF-alpha produced significant increases in AKT activation, leading to actin cytoskeletal reorganization in a redox sensitive manner (52). AKT plays a role in cytoskeletal reorganization, which promotes invasion and migration of GBM cells (53) (Figure 1).

In summary, there is an intrinsic relationship between GBM, tissue microenvironment, and gliomagenesis. GBM microenvironment mainly comprises reactive immune-related cells, together with microglia, astrocytes, endothelial cells, pericytes, neural stem cells, and monocyte macrophages; the last are abundant together with microglia, constituting around 30% of tumor mass (54). These monocyte infiltrations into GBM result in a proinflammatory microenvironment that leads to alterations in redox homeostasis, promoting finally gliomagenesis (55). For this reason, some of the new therapeutic alternatives are focused on the development of new agents able to modulate redox status in the GBM microenvironment, alone or combined with agents that stimulate ROS production (56).

## Cellular Redox Environment

Cellular redox status is described as the net physiologic balance between inter-convertible oxidized and reduced equivalents within subcellular compartments that remain in dynamic equilibrium. Under normal physiological conditions, ROS are produced constantly during cellular respiration and mediates the stimulation of various signaling pathways according to environmental conditions (57). Mitochondria are the major active site of ROS production due to incomplete coupling of electrons and H^+^ with oxygen in the electron transport chain (58, 59). During electron transfer through the respiratory chain, mitochondria generate large portion of ROS, such as superoxide, hydroxyl radical, and hydrogen peroxide, into the matrix and intermembrane space (60). The formation of superoxide occurs *via* the transfer of a free electron to molecular oxygen. Complex I (NADH dehydrogenase) and III (ubisemiquinone) of the electron transport chain produce most of the superoxide (61, 62). Superoxide is catalyzed to H_2_O_2_ by manganese superoxide dismutase (MnSOD) in the mitochondrial matrix or copper/zinc-SOD (Cu/Zn-SOD) in the cytosol. Then, H_2_O_2_ is degraded to oxygen and water by the reaction with catalase, peroxiredoxin and GSH peroxidase (mitochondria). However, when the balance between oxidants and antioxidants is broken, superoxide can react with nitric oxide producing peroxynitrite (ONOO^−^), and H_2_O_2_ reacts with reduced transition metals giving hydroxyl radical. The consequence of high ROS contents and Ca^2+^ overloading is that the mitochondrial permeability transition (MPT) pore is open, which leads to disruption of mitochondrial membrane potential and release of cytochrome *c* and proapoptotic molecules, from the inner membrane space of mitochondria to the cytosol (63), increasing even more ROS production.

Another important organelle that can mediate the cellular redox homeostasis is the endoplasmic reticulum (ER), the major site of calcium storage, where the folding of proteins and formation of disulfide bonds occur. The lumen of ER, in contrast to the cytosol, has a highly oxidizing environment, which facilitates disulfide bond formation and prevents the aggregation or accumulation of unfolded proteins. With this process, ER contributes to 25% of ROS generated by cell (64, 65); the lumen of ER also contains high ratio of GSSG/GSH. Protein folding is a energetically demanding process where ATP is required and the conditions that alert this tightly regulated environment, as glucose deprivation and alteration in the oxidative phosphorylation (OXPHOS), can cause an imbalance in the ER protein folding, leading to accumulation of unfolded proteins in the ER lumen, condition named as ER stress. Accumulation of unfolded proteins in the ER provoke Ca^2+^ leakage into the cytosol, activating MPT pore, which affects mitochondrial membrane potential, leading to ATP depletion and increasing ROS production in the mitochondria (66, 67). Also, it is important to mention that mitochondria and ER are linking organelles due to their close proximity and their capability to modulate calcium levels in the cytosol, which initiates a sequence of events where oxidative stress increases and the redox homeostasis is lost (68).

Many other enzymes, such as NADPH oxidase (69), xanthine oxidase (47), α-ketoglutarate dehydrogenase complex, d-amino acid oxidases, and dihydrolipoamide dehydrogenase (70), and other flavoproteins also produce ROS along the normal metabolism, although in lower concentrations.

Reactive species of oxygen are beneficial for the cell in low concentrations and play a key in role signal transduction, enzyme activation, gene expression, and disulfide bond formation, during the folding of new proteins in the ER, and control of caspase activity during apoptosis (71). They also have a role in the normal functioning of immune response, proliferation of T cells and activation of immunological peptides, as well as in the response in the regulation of various cell activities. ROS keep under control the balance between self-renewal, proliferation, and differentiation of normal stem cells and progenitor cells, either in hematopoietic or neuronal compartments (72, 73).

Reactive species of oxygen production is inhibited by endogenous antioxidants as SOD, catalase (CAT), glutathione, glutathione peroxidase (GPx), and glutathione reductase (Table 1), among others, which can prevent the generation of scavenging molecules and inactivate the already formed oxidants. Glutathione (GSH) redox cycle and thioredoxin represent the major cellular redox buffer (74, 75). In this context, GSH is a relevant low-molecular-weight thiol in cells, essential for normal redox signaling (76). During the oxidative stress, its oxidized form (GSSG) may accumulate, leading to deleterious consequences for metabolic regulation, cellular integrity and homeostasis. GSH status is maintained in reduced state by GSH peroxidase and GSSH reductase system, which are coupled to the oxidized and reduced nicotinamide dinucleotide phosphate (NADP/NADPH) redox pair. These antioxidants provide essential information on cellular redox state and affect the expression of genes associated with stress responses to maximize homeostasis.

**Table 1:** **Redox components alteration in various glioblastoma cell lines**.

Organelle	Antioxidant present	Reactive specie produced in normal conditions
Mitochondria	MnSOD	${\text{O}}_{2}^{\;•-}$, H_2_O_2_, O_2_, OH^⋅^
	Glutathione peroxidase	
	Glutathione reductase	
	Catalase	
	Quinones (coenzymes Q)	
	GSH	
	NADH	
	Thioredoxin	
ER	Glutathion (GSH)	O_2_, H_2_O_2_, OH^⋅^
	Cu/Zn-superoxide	
	Thioredoxin	
	Glutaredoxin	
	Peroxiredoxin	
	Endoplasmic reticulum oxidase	
	Protein disulfide isomerase	
	Quinones (coenzymes Q)	
Golgi	Quinones (coenzymes Q)	${\text{O}}_{2}^{\;•-}$, H_2_O_2_, OH^⋅^
	Cu, Zn-SOD	
	Transferrine	
Peroxisomes	Catalase	${\text{O}}_{2}^{\;•-}$, H_2_O_2_
	NADH	
	FAD	
	Cytochrome *b*	
	Ubiquinone	
Cytosol	Cu/Zn-SOD	${\text{O}}_{2}^{\;•-}$, H_2_O_2_, OH^⋅^, O_2_
	Protein disulfide isomerase	
Chloroplast	Protein disulfide isomerase	${\text{O}}_{2}^{\;•-}$, H_2_O_2_, OH^⋅^
	Quinones (coenzymes Q)	
Nucleus	Glutathione and thioredoxin	${\text{O}}_{2}^{\;•-}$, H_2_O_2_, OH^⋅^

When the cellular redox homeostasis is disturbed and the balance between cellular pro-oxidants and antioxidants is broken in favor of pro-oxidants, the cell enters into oxidative stress. During oxidative stress, ROS [superoxide $\left({\text{O}}_{2}^{\;•-}\right)$, hydrogen peroxide (H_2_O_2_), hydroxyl radical (OH^⋅^)], reactive nitrogen species (RNS) [nitric oxide (NO) and peroxynitrite (ONOO^−^)], reactive sulfur species (RSS), and reactive chloride species (RCS) are produced (77). ROS can react with the most relevant macromolecules, such as DNA, RNA, proteins, and lipids (78), leading to cell damage and DNA alterations. DNA oxidation by these reactive species generates 8-hydroxy-2-deoxyguanosine, which may induce DNA mutations, generating mutagenesis and disruptions of genomic stability, in a process that enhances aging and cancer development (79). Those variations in ROS concentrations do not only affect genomic DNA but also produce alterations in DNA mitochondrial due to their proximity to the electron transport chain. As was mentioned before, depending on the ROS levels, different redox-sensitive factors are activated and distinct biological responses are produced. When ROS levels are low, Nrf2, a transcription factor considered as a ROS receptor in mammals, is activated, which involves the transactivation of gene coding for antioxidant enzymes (80). However, when the levels of ROS are high, perturbation occurs in calcium homeostasis leading to MPT pore perturbation, disruption of the electron transfer chain, lipase activation, induction of inflammatory response, trough the activation on NF-κB and AP-1, all these outcomes in apoptosis o necrosis (81).

The nervous tissue is particularly vulnerable to oxidative stress due its high demand for oxygen and its inefficient defense mechanisms against free radicals, together with a high concentration of metal ions (e.g., iron and copper) involved in redox reactions (82). This complicated scenery within CNS has been related to the development of neurodegenerative diseases such as Alzheimer’s and Parkinson and it seems possible to various tumors in the brain (83, 84). It has been described that other ROS generators also contribute to tumor development. In this context, NADPH oxidases activate redox signaling pathways leading to angiogenesis (85, 86); mutant Ras can modulate NADPH causing increase in ROS, DNA damage, and cell transformations (87, 88); thus, cells that overexpress the oncogenic Ras display increased mitochondrial mass and ROS accumulation.

## Redox Environment Alteration in Glioblastoma

Cells are constantly exposed to oxidant damage, either by exogenous (X or γ rays, α particles, oxidant products, or UV) or by endogenous agents (cell signaling, metabolic and inflammatory processes) (89–92). These agents induce DNA changes producing complex DNA damage, i.e., double-strand DNA breaks (DSBs) and non-DSB generates clustered DNA lesions (OCDLs). Even low doses of IR (0.03 Gy) are enough to induce DNA mutations (93–95). Chronic exposure to viral infections can be also a source of free radicals that decrease the production of antioxidant enzymes such as catalase, glutathione peroxidase, glutathione reductase, as well as high levels of hydroxyl radicals (96).

Reactive nitrogen species and ROS are the main effectors of oxidant damage (97); although ROS have relatively brief periods of life, they can induce local DNA damage. H_2_O_2_ is another oxidant species with a longer period of life that may induce cell damage in distant sites to its niche (98). It is known that ROS have specific targets such as *OH·y ^1^O_2_, which react with DNA and proteins, while H_2_O_2_ use Fe^2+^ to promote the Fenton reaction (99).

The most common modifications induced by ROS in the DNA are -oxo-7,8-dihydroguanine (8-oxoGua) and 2,6-diamino-4-hydroxy-5-formamidopyrimidine, which lead to the production of apurinic/apyrimidinic (abasic) DNA sites, to oxidized purines and pyrimidines and to single-strand DNA breaks (SSBs) and DSBs (89, 100), finally inducing genetic instability and the possible emergence of brain tumors (89).

Cellular redox imbalance has been found in GBM (Table 2). ROS can exert different effects according to the basal metabolic rate of the cells. The CNS has high metabolic activity and fatty acids content, reasons why is particularly sensible to oxidant damage by ROS. Within CNS, astrocytes and neurons have antioxidant systems such as the GSSG–GSH system that protects these cells of oxidant damage; however, the expression of mRNA for SOD and catalase enzyme is high in astrocytes. These differences in the expression of antioxidant enzymes make astrocytes particularly sensitive to damage induced by ROS, leading to genetic instability when the redox balance is lost.

**Table 2:** **Redox therapies designed against GBM**.

Glioblastoma cellular line	Anticancer compound	Redox effects	Reference
U87–MG	SIRT6 (deacetylase)	↑ Apoptosis	(101)
T98G		↓ Oxidative stress	
		↓ JAK2/STAT3 signaling pathway	

U87MG	Chloroquine	↓ Cell viability (75–200 μM) 48 h	(102)
U343MG		↓ Mitochondrial membrane potential (50–200 μM, 12–24 h)	
U138MG		↑ Mitochondrial ${\text{O}}_{2}^{\;•-}$ production (50 μM)	
U251MG		↑ ROS production (50 μM)	
A172		150 μM	(103)
		↑ Apoptosis	
		↑ Nitric oxide	
		↑ ROS	
		↓ GSH levels	
		↑ GSH peroxidase activity	
		↑ GSH S-transferase	
C6 glioma cells		30–300 μM	(101)
		↑ iNOS expression	
		↑ NO production	

C6 glioma cells	AGEs (advanced glycosylation end products) (30–300 μg/ml)	↑ iNOS	(104)
		↑ Nitric oxide synthase expression	

C6 glioma cells	t-BOOH (tertiary-butylhydroperoxide)	↑ ROS generation	(105)
		↑ Lipid peroxidation	
		↓ GSH levels	
		↑ Ca^2+^ influx	

C6 glioma cells	OGD (oxygen-glucose deprivation)	↑ ROS generation	(106, 107)
		↑ Intracellular Ca^2+^	
		↑ Depolarization of mitochondrial inner membrane potential	

T98G	Quercetin (50 μM), temozolomide (50 μM), individual and in combination	↓ Mitochondrial membrane potential	(108)
C6 glioma cells	Quercetin (25 and 50 μM)	↑ ROS generation	(109)
	Rutin (25 and 50 μM)	↓ Cell viability	

U87MG	EGCG (epigallocatechin-3-gallate)	25, 50, and 100 μM	(110)
		↑ ROS generation	
		↓ Mitochondrial membrane potential	
T98G		50 μM	(111)
U87MG		↑ Apoptosis	
		↑ ROS production activation of the redox-sensitive c-Jun N-terminal kinase 1 pathway	
		↓ Mitochondrial membrane potential	
		↓ Cell viability	

8401 GBM cells	PEITC (phenethyl isothiocyanate)	↑ ROS generation	(112)
		Mitochondrial dysfunction	

T98G cells	Gambogic acid (200–400 nM)	↑ ROS generation	(113)
		↑ Apoptosis	

U87MG	Artocarpesin (106 μM), cycloartocarpesin (50 μM), and isobavachalcone (25 μM)	↑ ROS generation	(114)
		↓ Mitochondrial membrane potential	

GSC11	Serum	↑ Mitochondrial ROS generation	(115)
GSC23		↑ SOD expression	
GBM3752		↑ Catalase expression	
		↓ GSH levels	

U87MG	Pt-1-DMCa (platinum analog)	↑ ROS generation	(116)
		↑ Apoptosis	

GBM3752	Temozolomide, demethoxycurcumin	↑ ROS generation	(117)
		↑ Apoptosis	
		↓ JAK/STAT3 signaling pathway	

U251 and U87	Arecaidine propargyl ester (25–100 μM)	↑ ROS generation	(118)
		↑ SOD expression	
		↑ Apoptosis	

GSC 387 and 3832	Cannabidiol (3.5 μM)	↑ ROS generation	(119)
		↓ Cell viability	

U87 (human)	Oligomeric procyanidins (30–100 μg/ml)	↑ ROS generation	(120)
C6 (rat)		↓ Mitochondrial membrane potential	
		↓ Cell viability	

U87	Alantolactone (10–60 μM)	↓ GSH	(121)
U373		↑ ROS production	
LN229		↓ Mitochondrial transmembrane potential	

GL15	Bromopyruvate	↓ Mitochondrial potential	(122)
		↓ MTT	
		↓ ATP	
		↑ Apoptosis	
		↑ ROS production	

D-54 MG	Manganese porphyrin	↓ ROS production	(123)
D-245 MG		↓ RNS production	
D-256 MG		↑ SOD expression	
D-456 MG			

T98G	Apigenin (50 μM), epigallocatechin (50 μM), and Genistein (50 μM)	↑ Apoptosis	(111)
U87MG		↑ ROS production activation of the redox-sensitive c-Jun N-terminal kinase 1 pathway	
		↓ Mitochondrial membrane potential	
		↓ Cell viability	

LN229	Kaempferol (50 μmol/L)	↑ Apoptosis	(124)
U87MG		↑ ROS production
T98G		↓ Cell viability
		↓ SOD-1 expression (superoxide dismutase)
		↓ TRX-1 (thioredoxin)
		↓ Mitochondrial membrane potential

U87	PENAO (4-(N-(S-penicillaminylacetyl) amino) phenylarsonous acid) (0–10 μM), DCA (0–50 mM) alone and combination	↓ Cell viability	(125)
U251		↑ Apoptosis	
LN229		↑ Depolarized mitochondria	
		↑ ROS production	
		↑ Mitochondrial ROS production	
		↓ Oxygen consumption rate (PENAO)	
		↑ Oxygen consumption rate (DCA)	
		= Oxygen consumption rate (combination)	
		↑ Extracellular acidification rate (PENAO)	
DBTRG		↓ Extracellular acidification rate (DCA and combination)	

GBM cells	DCA	↑ Depolarized mitochondria	(126)
		↑ ROS production	
		↑ Mitochondrial ROS production	
		↑ Apoptosis	
		↑ Oxidative phosphorylation	

U-13898	Ascorbic acid (5–100 mmol/L)	↓ Cell viability	(127)
U-87		↑ ROS production	
U-251		↑ H_2_O_2_ production	

T98G	Xanthohumol	↓ Cell viability	(128)
		↑ Apoptosis	
		↑ Intracellular ROS production	

T98G	Berberine (0–200 μg/ml)	↓ Cell viability	(129)
		↑ ROS production	
		↑ Intracellular Ca^2+^	
		↑ Endoplasmic reticulum	

T98G	Buthionine sulfoximine	↓ GSH	(130, 131)
U87MG		↓ Cell viability	

Cancer cells show high basal levels of ROS, necessary for their increased proliferative rate (48). Recent studies have shown that high levels of ROS in cancer cells are the result of increased basal metabolic activity, mitochondrial dysfunction, due to hypoxia or mitophagy, peroxisomes activity, uncontrolled growth factors of cytokine signaling, oncogene activity, as well as enhanced activity of known ROS sources, such as NADPH oxidase, cyclooxygenases, or lipoxygenases (132–134) in cancer cells. The alteration on redox homeostasis is involved in the beginning, progression and regression of neoplasm. As was mentioned, reduction-oxidation (redox) reactions that generate ROS, including ${\text{O}}_{2}^{\;•-}$, H_2_O_2_, and OH^⋅^, have been reported as important chemical mediators in the regulation of signal transduction. Due to the high levels of ROS, cancer cells also stimulate the antioxidant system, such as MnSOD, catalase, and glutathione peroxidase, to eliminate ROS (135) (Table 1). Conversely, ROS can also stimulate intracellular signal events, promoting activation in tumor cells, due to the capacity to stimulate kinases and small G proteins such as c-Src, Ras, and ERK1/2 (136, 137), leading to cell proliferation. In the same way, negative regulation of SOD-1, as well as the addition of TNF-α to GBM cells, generate increase in the ROS production, leading to SOD-1 decline in a exposure time-dependent manner, and to rise the phosphorylation of AKT in a redox status-dependent manner, which induces the reorganization of the actin cytoskeleton (52).

Due to the action of flavoproteins, malignant cells constitutively produce high H_2_O_2_ concentrations. These chronic amounts of H_2_O_2_ are enough to induce DNA damage without apoptosis induction, nor genetic instability in the nucleus and mitochondrial DNA, in a concentration/intracellular dependent manner. Tumor resistance and malignancy may occur when those punctual mutations are generated in critical genes that control metabolism and cell cycle (138). Besides, high amounts of H_2_O_2_ activate several pathways, acting as “second messenger,” increasing the expression of oxidant stress factors and producing a rise in the expression of antioxidant enzymes that protect malignant cells from apoptosis induction (48, 118, 139).

Additionally, the redox/Fyn/c-Cbl (RFC) pathway plays a key role in the activation of growth factors, involved in cell proliferation. In the RFC pathway, cellular oxidation causes sequential activation of Fyn kinase and c-Cbl ubiquitin ligase, in the oligodendrocyte/type-2 astrocyte progenitor cells (O-2A/OPCs). These activations guide to ubiquitylation and degradation of c-Cbl’s protein targets, such as growth factor and EGFRs (140), the C-Met hepatocyte growth factor receptor (HGFR) (140), and the insulin-like growth factor-I receptor (141), among others. In this context, GBM treatment with the antineoplastic 1,3-bis(2-chloroethyl)-1-nitrosourea (BCNU) (carmustine) may induce DNA crosslinks, inhibition of glutathione reductase, and increase of the intracellular oxidative status, events that bring as consequence the pathway activation without c-Cbl phosphorylation and without reductions in EGFR contents (a c-Cbl target frequently overexpressed in GBMs and other cancers) (142, 143). In GBM cells, the phosphorylation of c-Cbl in response to BCNU is prevented. This failure causes c-Cbl activation and decreases EGFR levels in GBM cells due to the c-Cbl sequestration by Cool-1 protein (144) (Figure 1).

Moreover, the high-mobility group 1 (HMGB1) molecule has been associated with progression, invasion, and tumor metastasis; it is abundantly expressed in several tumors and in undifferentiated cells (145). HMGB1 is a classic DAMP released by necrotic cells and secreted by monocytes, macrophages, and dendritic cells (DCs) (146, 147). HMGB1 functions as a sensor of intracellular oxidative status, being released after oxidation of cysteine residues; it induces DNA damage by ROS and promotes genomic instability in neoplastic cells (148–150). TLR2, TLR4, TLR9 and RAGE are receptors expressed in macrophages that can bind to HMGB1 and signaling NF-κB, resulting in the release of pro-inflammatory molecules (151). These tumor-resident macrophages could sustain the inflammatory environment inside the tumor, together with neutrophils, contributing to enhance oxidative status trough the release of high amounts of ROS and activation of NOX2 in response to several DAMPs (for instance, HMGB1; Figure 1) (151). Tumor cells take advantage of this inflammatory environment to develop, proliferate and produce new tumor endothelial cells to sustain angiogenesis, to release cytokines, growth factors, extracellular matrix-degrading enzymes and angiogenic factors, such as vascular endothelial growth factor (VEGF), Bv8, and MMP9 (152). Besides, tumor cells inhibit the specific immune response (T cell activity) trough IL-10, TGF-β, and ROS production (29, 153, 154).

## Redox Therapeutics on Glioblastoma

Multitude of active substances has been tried for therapy of GBM. As described above, oxidative environment supports the survival of GBM cells inducing healthy cells to produce antioxidant enzymes, such as catalase and SOD, to decrease the raised levels of ROS (155, 156) (Table 1). Additionally, this environment leads to inactivate the tumor suppressor protein p53, enabling tumor cells to escape apoptosis (157), therefore inhibiting the therapeutic effects of radio/chemotherapy (158). As the redox environment plays an important role in the initiation, progression, and regression of a tumor, new alternative redox therapies have been investigated. Here, we described some of these redox therapies designed against GBM (Table 2).

Recently, Singer and coworkers showed that cannabidiol (a cannabinoid) possess anti-tumoral effect in 3832 and 387 GBM cell lines both *in vitro* and *in vivo*. The antitumoral effect is partially attributed to ROS production *in vitro*. The cannabinoid inhibited glioma stem cells viability through ROS production and this effect was abolished by the co-incubation with vitamin E. Additionally, cannabidiol inhibited GBM progression *in vivo* and increased survival of GBM-bearing mice. However, a subset of glioma stem cells became adapted by activating an extended antioxidant cellular response; in part due to NRF2 transcriptional network as well as to redox system Xc catalytic subunit xCT (SLC7A11).

One of the most important participants in the variability of GBM cells is glucose metabolism, which represents the main route to support their growth, and it is related with chemoresistance. This glycolytic ability is characterized by a shift from OXPHOS toward aerobic glycolysis as the main source of ATP production; this effect is commonly called the Warburg effect (159) and mitochondrial functions are partially activated in these cells (160). Due to the importance of glycolysis for GBM cells, several blockers of this metabolic pathway have been tested as anticancer agents, *in vitro* and *in vivo* (161–163); however, only minor positive results have been obtained. In this context, dichloroacetate, a pyruvate dehydrogenase kinase (PDK) inhibitor, reverses the Warburg effect by a shift from glycolysis to mitochondrial oxidation, inducing a cytotoxic effect in various human malignant cell lines (164, 165). The target enzyme of dichloroacetate is highly expressed in GBM cells, inducing cell cycle arrest in G2/M phase of GBM cell cultures, however, it had not effect on non-cancerous cells; dichloroacetate also increases ROS production due to pyruvate participation in mitochondrial oxidation, depolarizes mitochondria, and induces apoptosis in glioblastoma cells. Additionally, the efficacy of radiotherapy was enhanced by dichloroacetate in glioblastoma cells, both strategies worked synergistically, *in vivo* and *in vitro*, to elevate mitochondrial ROS levels and γ-H2AX (a hallmark of DNA damage) in GBM cells. Shen and coworkers also observed that the combination of dichloroacetate with temozolamide increases the apoptosis observed with temozolamide alone in GBM stem cells (125).

Mitochondria are other components that play an important role in glioblastoma cells. They participate in a wide array of cellular processes, particularly confer resistance to apoptosis, considering that glycolysis and energetic metabolism are common factors in glioblastoma. Shen and coworkers have shown that dichloroacetate, restores mitochondrial activity and combined with a mitochondrial toxin enhances synergistically the cytotoxicity of GBM cells. The mechanisms by which these agents lead to apoptosis involve ROS production, considering that the simultaneous incubation with an antioxidant decreased the number of apoptotic cells, as was observed by co-incubation with inhibitors of glycolysis (125). Another factor that might play a role in twitching aerobic glycolysis back to OXPHOS is rapamycin (mTOR), which is overexpressed in many human tumors (166). mTOR is a critical regulator of cell proliferation; its dysfunction can transform normal cells into tumor like-cells (167) and switch the energetic metabolism from OXPHOS to aerobic glycolysis (168).

Muscarinic receptors are also expressed in glioblastoma cells; the M2 subtype appears relevant for their proliferation and survival (169). The activation of M2 receptors by arecaidine causes an arrest of the cell cycle and consequent apoptosis (169). These effects, induced by arecaidine, appear to be mediated by ROS production as the co-incubation with the antioxidant *N*-acetyl-l-cysteine decreases ROS levels and the apoptotic index in U87MG and U251MG GBM cell lines. Additionally, SIRT1, a member of the sirtuin family, and able to activate stress defenses and DNA repair machinery, increases its expression after treatment with arecaidine. The MnSOD expression is also augmented with this activator of M2 receptors. The rise in apoptosis caused by arecaidine could be explained by the simultaneous increase of SIRT1 expression, protein that induces apoptosis when the stress becomes chronic or when the cell damage appears to be irreversible (118).

Apigenin and other flavonoids induce apoptosis in human glioblastoma T98G and U87MG cells through various pathways: increase of ROS production, phosphorylation of p38 MAPK, activation of the redox-sensitive c-Jun N-terminal kinase 1, downregulated expression of the anti-apoptotic protein Bcl-2, and activation of the anti-apoptotic kinase Akt, as well as by suppressing the expression of inflammatory factors (NF-κB and COX-2) and activation of death receptor and mitochondrial pathways (111). Other studies show that quercetin (a flavonoid) possess anticancer effects, inhibiting significantly the proliferation of U373MG cells in a concentration-dependent manner, by cell death through apoptosis, as is evidenced by the increased number of cells in the sub-G1 phase (170). Also, when quercetin was combined with temozolomide (TMZ), the current chemotherapeutic agent used in T98G GBM cells treatment, induced apoptosis which correlated with caspase 3 and 9 activation, cytochrome *c* release from the mitochondria and decrease in the mitochondrial membrane potential (108, 171, 172).

Recently, it was reported that melatonin inhibits HIF-1α protein and suppress the expression of matrix metalloproteinase 2 (MMP-2) and VEGF by means of its antioxidant activity, reducing the invasion and migration mediated by hypoxia, of U251 and U87 glioblastoma cells (173). Additionally, alantolactone, a sesquiterpene lactone compound, induces GSH depletion, inhibits growth and triggers apoptosis of glioblastoma cells. These effects induced by alantolactone can be directly related to ROS generation due to *N*-acetyl-l-cysteine – an antioxidant that prevents apoptosis and GSH depletion (121). In addition, GSH synthesis inhibitors potentiate the TMZ effect (174).

Another strategy to modulate the redox environment in GBM is the use of buthionine sulfoximine (BSO), a potent blocker of glutathione synthesis through inhibition of γ-glutamyl-cysteine synthetase. BSO shows to enhance the cytotoxic effect of various drugs in cancer cell (175–177). Specifically in human glioblastoma cell lines (T98G, U87MG), BSO increased their sensitivity against platinum compounds (130) and hydrogen peroxide (131). BSO represents a viable strategy to explore in the future for glioblastoma therapy, considering that astrocytes have higher contents of GSH and GSH intermediates than neurons (178, 179), but also because glioblastoma cell lines (T98G, U87MG) possess more intracellular GSH than other malignant cells as human myelogenous leukemic cells (HL-60).

Ascorbate (vitamin C) has also been used as an anticancer treatment. Studies made in LN18 GBM cell line, mouse astrocytoma cell line GL261, and untransformed astrocyte cell line C8D1A have shown that ascorbate increases radiation sensitivity in a dose-dependent manner and interferes with the cell cycle progression (180). Another study in human cancer cells showed that 55% of the human cancer cell lines were susceptible to the oxidative stress mediated by ascorbic acid through the production of hydrogen peroxide (127). Various agents, such as the antiglycolytic bromopyruvate, xanthohumol, and berberine, induce cell death in glioblastoma cell lines through ROS production (Table 2) (122, 128, 129). All the drugs described here involve an alternative strategy to modulate redox in the GBM environment. However, most of these drugs give insights about the involved mechanism and offer novel routes to facilitate discovery cancer-specific therapies.

## Concluding Remarks

There are various theories about the origin of GBM; one of them indicates that inflammatory processes, together with redox alterations are common factors in the origin of several neoplasias, generating alterations that promote an abnormal circle between oxidant environment, chromosomal and mitochondrial instability and inflammation, which are factors that contribute to the malignancy and proliferation of GBM. Little is known about the direct influence of ROS in the intra and extra signaling pathways of GBM cells and how these substances participate in the cellular metabolism, contributing in a high degree in proliferation and resistance. Is important to develop new therapeutic alternatives focused on the peculiar cellular redox environment of gliomagenesis; these novel approaches might increase the efficacy, supporting therapeutic interventions focused to improve the cellular redox homeostasis and induce apoptosis of abnormal cells, in order to reduce their proliferation rate and provoke differentiation.

## Author Contributions

All the authors provided the information of Glioblastoma, besides to help with the search of information of reactive species of oxygen and their role in the tumor microenvironment.

## Conflict of Interest Statement

The authors declare that the research was conducted in the absence of any commercial or financial relationships that could be construed as a potential conflict of interest.

## Abbreviations

AGEs, advanced glycosylation end products; AKT, protein kinase b; ATP, adenosine triphosphate; BCNU, bis-chloroethylnitrosourea (carmustine); CAT, catalase; CDK4, cyclin-dependent kinase 4; CMV, cytomegalovirus; CNS, central nervous system; CSC, carcinogenic stem cells; DAMPs, damage-associated molecular patterns; DCs, dendritic cells; DCA, dichloroacetate; DNA, deoxyribonucleic acid; EGCG, epigallocatechin-3-gallate; EGFR, epidermal growth factor receptor; GBM, glioblastoma multiform; γ-H2AX, hallmark of DNA damage; GPx, glutathione, glutathione peroxidase; GSH, reduced glutathione; GSSG, oxidized glutathione; H^+^, hydrogen; H_2_O_2_, hydrogen peroxide; HGFR, hepatocyte growth factor receptor; HMGB1, high-mobility group 1; IL, interleukin; MAPK, mitogen-activated protein kinase; MMP, matrix metalloproteinase; MnSOD, manganese superoxide dismutase; mTOR, mammalian target of rapamycin; NADP, oxidized nicotinamide dinucleotide phosphate; NADPH, reduced nicotinamide dinucleotide phosphate; NF-κB, nuclear factor kB; NO, nitric oxide; O2^⋅−^, superoxide; O-2A/OPCs, oligodendrocyte/type-2 astrocyte progenitor cells; OGD, oxygen-glucose deprivation; OH^⋅^, hydroxyl radical; ONOO^−^, peroxynitrite; OXPHOS, oxidative phosphorylation; PAMPs, pathogen-associated molecular patterns; PDGFA/PDGFRα, platelet-derived growth factor A receptor-α; PDK, pyruvate dehydrogenase kinase; PEITC, phenethyl isothiocyanate; PENAO, 4-(N-(S-penicillaminylacetyl) amino) phenylarsonous acid; PI3K, phosphoinositide 3-kinase; Pt-1-DMCa, platinum analog; PTEN, phosphatase and tensin homolog; RB, retinoblastoma protein; RCS, reactive chloride species; RFC, redox/Fyn/c-Cbl; RNA, ribonucleic acid; RNS, reactive nitrogen species; ROS, reactive oxygen species; RSS, reactive sulfur species; SOD, superoxide dismutase; SVZ, subventricular zone; t-BOOH, tertiary-butylhydroperoxide; TGF, transforming growth factor; TLR, toll-like receptor; TNF-α, tumor necrosis factor; VEGF, vascular endothelial growth factor; VEGFR, vascular endothelial grow factor receptor.

## References

[1] Lopez-Gonzalez MA Sotelo J. Brain tumors in Mexico: characteristics and prognosis of glioblastoma. Surg Neurol (2000) 53(2):157–62. 10.1016/S0090-3019(99)00177-9 10713194

[2] Ostrom QT Gittleman H Farah P Ondracek A Chen Y Wolinsky Y CBTRUS statistical report: primary brain and central nervous system tumors diagnosed in the United States in 2006-2010. Neuro Oncol (2013) 15(Suppl 2):ii1–56. 10.1093/neuonc/not151 24137015 PMC3798196

[3] Piccirillo SG Sottoriva A Watts C The role of sub-ventricular zone in gliomagenesis. Aging (Albany NY) (2015) 7(10):738–9. 10.18632/aging.100823 26527608 PMC4637196

[4] Ohgaki H Kleihues P. Genetic pathways to primary and secondary glioblastoma. Am J Pathol (2007) 170(5):1445–53. 10.2353/ajpath.2007.070011 17456751 PMC1854940

[5] Scherer HJ A critical review: the pathology of cerebral gliomas. J Neurol Psychiatry (1940) 3(2):147–77. 10.1136/jnnp.3.2.147 21610973 PMC1088179

[6] Buckner JC. Factors influencing survival in high-grade gliomas. Semin Oncol (2003) 30(6 Suppl 19):10–4. 10.1053/j.seminoncol.2003.11.031 14765378

[7] Alcantara Llaguno SR Chen J Parada LF. Signaling in malignant astrocytomas: role of neural stem cells and its therapeutic implications. Clin Cancer Res (2009) 15(23):7124–9. 10.1158/1078-0432.CCR-09-0433 19934302 PMC2787668

[8] Chen J Li Y Yu TS McKay RM Burns DK Kernie SG A restricted cell population propagates glioblastoma growth after chemotherapy. Nature (2012) 488(7412):522–6. 10.1038/nature11287 22854781 PMC3427400

[9] Zhu Y Harada T Liu L Lush ME Guignard F Harada C Inactivation of NF1 in CNS causes increased glial progenitor proliferation and optic glioma formation. Development (2005) 132(24):5577–88. 10.1242/dev.02162 16314489 PMC2760350

[10] Piccirillo SG Spiteri I Sottoriva A Touloumis A Ber S Price SJ Contributions to drug resistance in glioblastoma derived from malignant cells in the sub-ependymal zone. Cancer Res (2015) 75(1):194–202. 10.1158/0008-5472.CAN-13-3131 25406193 PMC4286248

[11] Pflaum J Schlosser S Muller M p53 Family and cellular stress responses in cancer. Front Oncol (2014) 4:285 10.3389/fonc.2014.00285 25374842 PMC4204435

[12] Reardon DA Ligon KL Chiocca EA Wen PY. One size should not fit all: advancing toward personalized glioblastoma therapy. Discov Med (2015) 19(107):471–7. 26175405

[13] Bae YS Kang SW Seo MS Baines IC Tekle E Chock PB Epidermal growth factor (EGF)-induced generation of hydrogen peroxide. Role in EGF receptor-mediated tyrosine phosphorylation. J Biol Chem (1997) 272(1):217–21. 10.1074/jbc.272.1.217 8995250

[14] Miller EW Tulyathan O Isacoff EY Chang CJ. Molecular imaging of hydrogen peroxide produced for cell signaling. Nat Chem Biol (2007) 3(5):263–7. 10.1038/nchembio871 17401379

[15] Li J Yen C Liaw D Podsypanina K Bose S Wang SI PTEN, a putative protein tyrosine phosphatase gene mutated in human brain, breast, and prostate cancer. Science (1997) 275(5308):1943–7. 10.1126/science.275.5308.1943 9072974

[16] Myers MP Pass I Batty IH Van der Kaay J Stolarov JP Hemmings BA The lipid phosphatase activity of PTEN is critical for its tumor supressor function. Proc Natl Acad Sci U S A (1998) 95(23):13513–8. 10.1073/pnas.95.23.13513 9811831 PMC24850

[17] Kwon J Lee SR Yang KS Ahn Y Kim YJ Stadtman ER Reversible oxidation and inactivation of the tumor suppressor PTEN in cells stimulated with peptide growth factors. Proc Natl Acad Sci U S A (2004) 101(47):16419–24. 10.1073/pnas.0407396101 15534200 PMC534546

[18] Lee SR Yang KS Kwon J Lee C Jeong W Rhee SG. Reversible inactivation of the tumor suppressor PTEN by H_2_O_2_. J Biol Chem (2002) 277(23):20336–42. 10.1074/jbc.M111899200 11916965

[19] Rao CB Dewan SK Auto-transplantation of teeth. Clinical experiences with auto-transplantation of developing mandibular third molars to first and second molar sites. J Indian Dent Assoc (1974) 46(10):391–6. 4532122

[20] Hussain SP Amstad P He P Robles A Lupold S Kaneko I p53-induced up-regulation of MnSOD and GPx but not catalase increases oxidative stress and apoptosis. Cancer Res (2004) 64(7):2350–6. 10.1158/0008-5472.CAN-2287-2 15059885

[21] Tan M Li S Swaroop M Guan K Oberley LW Sun Y. Transcriptional activation of the human glutathione peroxidase promoter by p53. J Biol Chem (1999) 274(17):12061–6. 10.1074/jbc.274.17.12061 10207030

[22] Yoon KA Nakamura Y Arakawa H. Identification of ALDH4 as a p53-inducible gene and its protective role in cellular stresses. J Hum Genet (2004) 49(3):134–40. 10.1007/s10038-003-0122-3 14986171

[23] Macedo GS Lisboa da Motta L Giacomazzi J Netto CB Manfredini V Vanzin CS Increased oxidative damage in carriers of the germline TP53 p.R337H mutation. PLoS One (2012) 7(10):e47010. 10.1371/journal.pone.0047010 23056559 PMC3467233

[24] Magana-Maldonado R Manoutcharian K Hernandez-Pedro NY Rangel-Lopez E Perez-De la Cruz V Rodriguez-Balderas C Concomitant treatment with pertussis toxin plus temozolomide increases the survival of rats bearing intracerebral RG2 glioma. J Cancer Res Clin Oncol (2014) 140(2):291–301. 10.1007/s00432-013-1565-3 24337403 PMC11823771

[25] Orozco-Morales M Sanchez-Garcia FJ Golan-Cancela I Hernandez-Pedro N Costoya JA de la Cruz VP RB mutation and RAS overexpression induce resistance to NK cell-mediated cytotoxicity in glioma cells. Cancer Cell Int (2015) 15:57. 10.1186/s12935-015-0209-x 26146488 PMC4491266

[26] Lin YC Hou SC Hung CM Lin JN Chen WC Ho CT Inhibition of the insulin-like growth factor 1 receptor by CHM-1 blocks proliferation of glioblastoma multiforme cells. Chem Biol Interact (2015) 231:119–26. 10.1016/j.cbi.2015.01.016 25643584

[27] Fine HA. New strategies in glioblastoma: exploiting the new biology. Clin Cancer Res (2015) 21(9):1984–8. 10.1158/1078-0432.CCR-14-1328 25670220

[28] Yin H Zhou Y Wen C Zhou C Zhang W Hu X Curcumin sensitizes glioblastoma to temozolomide by simultaneously generating ROS and disrupting AKT/mTOR signaling. Oncol Rep (2014) 32(4):1610–6. 10.3892/or.2014.3342 25050915

[29] Lahmar Q Keirsse J Laoui D Movahedi K Van Overmeire E Van Ginderachter JA Tissue-resident versus monocyte-derived macrophages in the tumor microenvironment. Biochim Biophys Acta (2015) 1865:23–34. 10.1016/j.bbcan.2015.06.009 26145884

[30] Bondy ML Scheurer ME Malmer B Barnholtz-Sloan JS Davis FG Il’yasova D Brain tumor epidemiology: consensus from the Brain Tumor Epidemiology Consortium. Cancer (2008) 113(7 Suppl):1953–68. 10.1002/cncr.23741 18798534 PMC2861559

[31] Jackson SP Bartek J. The DNA-damage response in human biology and disease. Nature (2009) 461(7267):1071–8. 10.1038/nature08467 19847258 PMC2906700

[32] Munoz-Espin D Serrano M. Cellular senescence: from physiology to pathology. Nat Rev Mol Cell Biol (2014) 15(7):482–96. 10.1038/nrm3823 24954210

[33] Xue W Zender L Miething C Dickins RA Hernando E Krizhanovsky V Senescence and tumour clearance is triggered by p53 restoration in murine liver carcinomas. Nature (2007) 445(7128):656–60. 10.1038/nature05529 17251933 PMC4601097

[34] Pateras IS Havaki S Nikitopoulou X Vougas K Townsend PA Panayiotidis MI The DNA damage response and immune signaling alliance: is it good or bad? Nature decides when and where. Pharmacol Ther (2015) 154:36–56. 10.1016/j.pharmthera.2015.06.011 26145166

[35] Rollison DE Helzlsouer KJ Alberg AJ Hoffman S Hou J Daniel R Serum antibodies to JC virus, BK virus, simian virus 40, and the risk of incident adult astrocytic brain tumors. Cancer Epidemiol Biomarkers Prev (2003) 12(5):460–3. 12750243

[36] Mitchell DA Xie W Schmittling R Learn C Friedman A McLendon RE Sensitive detection of human cytomegalovirus in tumors and peripheral blood of patients diagnosed with glioblastoma. Neuro Oncol (2008) 10(1):10–8. 10.1215/15228517-2007-035 17951512 PMC2600830

[37] Lehrer S Green S Ramanathan L Rosenzweig KE Rendo A Virology of malignant brain tumours. Eur Assoc NeuroOncol Mag (2013) 3(1):2 10.14791/btrt.2015.3.2.65

[38] Lehrer S Green S Rendo A Rosenzweig KE Measles may be a risk factor for malignant brain tumors. Brain Tumor Res Treat (2015) 3(2):65–7. 10.14791/btrt.2015.3.2.65 PMC4642282 26605259

[39] Tsai SY Segovia JA Chang TH Morris IR Berton MT Tessier PA DAMP molecule S100A9 acts as a molecular pattern to enhance inflammation during influenza A virus infection: role of DDX21-TRIF-TLR4-MyD88 pathway. PLoS Pathog (2014) 10(1):e1003848. 10.1371/journal.ppat.1003848 24391503 PMC3879357

[40] Li G Tang D Lotze MT Menage a trois in stress: DAMPs, redox and autophagy. Semin Cancer Biol (2013) 23(5):380–90. 10.1016/j.semcancer.2013.08.002 23994764 PMC4085481

[41] Piccinini AM Midwood KS. DAMPening inflammation by modulating TLR signalling. Mediators Inflamm (2010) 2010:672395. 10.1155/2010/672395 20706656 PMC2913853

[42] Takaoka A Hayakawa S Yanai H Stoiber D Negishi H Kikuchi H Integration of interferon-alpha/beta signalling to p53 responses in tumour suppression and antiviral defence. Nature (2003) 424(6948):516–23. 10.1038/nature01850 12872134

[43] Escamilla-Tilch M Filio-Rodriguez G Garcia-Rocha R Mancilla-Herrera I Mitchison NA Ruiz-Pacheco JA The interplay between pathogen-associated and danger-associated molecular patterns: an inflammatory code in cancer? Immunol Cell Biol (2013) 91(10):601–10. 10.1038/icb.2013.58 24100386

[44] Youn JH Oh YJ Kim ES Choi JE Shin JS. High mobility group box 1 protein binding to lipopolysaccharide facilitates transfer of lipopolysaccharide to CD14 and enhances lipopolysaccharide-mediated TNF-alpha production in human monocytes. J Immunol (2008) 180(7):5067–74. 10.4049/jimmunol.180.7.5067 18354232

[45] Kim T Kim TY Song YH Min IM Yim J Kim TK. Activation of interferon regulatory factor 3 in response to DNA-damaging agents. J Biol Chem (1999) 274(43):30686–9. 10.1074/jbc.274.43.30686 10521456

[46] Moiseeva O Mallette FA Mukhopadhyay UK Moores A Ferbeyre G. DNA damage signaling and p53-dependent senescence after prolonged beta-interferon stimulation. Mol Biol Cell (2006) 17(4):1583–92. 10.1091/mbc.E05-09-0858 16436515 PMC1415317

[47] Aggarwal BB. Signalling pathways of the TNF superfamily: a double-edged sword. Nat Rev Immunol (2003) 3(9):745–56. 10.1038/nri1184 12949498

[48] Szatrowski TP Nathan CF. Production of large amounts of hydrogen peroxide by human tumor cells. Cancer Res (1991) 51(3):794–8. 1846317

[49] Wallach D Varfolomeev EE Malinin NL Goltsev YV Kovalenko AV Boldin MP. Tumor necrosis factor receptor and Fas signaling mechanisms. Annu Rev Immunol (1999) 17:331–67. 10.1146/annurev.immunol.17.1.331 10358762

[50] Liang J Slingerland JM. Multiple roles of the PI3K/PKB (Akt) pathway in cell cycle progression. Cell Cycle (2003) 2(4):339–45. 10.4161/cc.2.4.433 12851486

[51] Radeff-Huang J Seasholtz TM Chang JW Smith JM Walsh CT Brown JH. Tumor necrosis factor-alpha-stimulated cell proliferation is mediated through sphingosine kinase-dependent Akt activation and cyclin D expression. J Biol Chem (2007) 282(2):863–70. 10.1074/jbc.M601698200 17114809

[52] Ghosh S Tewari R Dixit D Sen E. TNFalpha induced oxidative stress dependent Akt signaling affects actin cytoskeletal organization in glioma cells. Neurochem Int (2010) 56(1):194–201. 10.1016/j.neuint.2009.10.003 19836430

[53] Qian Y Zhong X Flynn DC Zheng JZ Qiao M Wu C ILK mediates actin filament rearrangements and cell migration and invasion through PI3K/Akt/Rac1 signaling. Oncogene (2005) 24(19):3154–65. 10.1038/sj.onc.1208525 15735674

[54] Becher OJ Hambardzumyan D Fomchenko EI Momota H Mainwaring L Bleau AM Gli activity correlates with tumor grade in platelet-derived growth factor-induced gliomas. Cancer Res (2008) 68(7):2241–9. 10.1158/0008-5472.CAN-07-6350 18381430

[55] Feng X Szulzewsky F Yerevanian A Chen Z Heinzmann D Rasmussen RD Loss of CX3CR1 increases accumulation of inflammatory monocytes and promotes gliomagenesis. Oncotarget (2015) 6(17):15077–94. 10.18632/oncotarget.3730 25987130 PMC4558137

[56] Manda G Isvoranu G Comanescu MV Manea A Debelec Butuner B Korkmaz KS. The redox biology network in cancer pathophysiology and therapeutics. Redox Biol (2015) 5:347–57. 10.1016/j.redox.2015.06.014 26122399 PMC4501561

[57] Jabs T. Reactive oxygen intermediates as mediators of programmed cell death in plants and animals. Biochem Pharmacol (1999) 57(3):231–45. 10.1016/S0006-2952(98)00227-5 9890550

[58] Drose S Brandt U. Molecular mechanisms of superoxide production by the mitochondrial respiratory chain. Adv Exp Med Biol (2012) 748:145–69. 10.1007/978-1-4614-3573-0_6 22729857

[59] Lenaz G. Mitochondria and reactive oxygen species. Which role in physiology and pathology? Adv Exp Med Biol (2012) 942:93–136. 10.1007/978-94-007-2869-1_5 22399420

[60] Wallace DC Brown MD Lott MT Mitochondrial DNA variation in human evolution and disease. Gene (1999) 238(1):211–30. 10.1016/S0378-1119(99)00295-4 10570998

[61] Boveris A Chance B. The mitochondrial generation of hydrogen peroxide. General properties and effect of hyperbaric oxygen. Biochem J (1973) 134(3):707–16. 10.1042/bj1340707 4749271 PMC1177867

[62] Turrens JF Boveris A. Generation of superoxide anion by the NADH dehydrogenase of bovine heart mitochondria. Biochem J (1980) 191(2):421–7. 10.1042/bj1910421 6263247 PMC1162232

[63] Kroemer G Dallaporta B Resche-Rigon M. The mitochondrial death/life regulator in apoptosis and necrosis. Annu Rev Physiol (1998) 60:619–42. 10.1146/annurev.physiol.60.1.619 9558479

[64] Malhotra JD Kaufman RJ Endoplasmic reticulum stress and oxidative stress: a vicious cycle or a double-edged sword? Antioxid Redox Signal (2007) 9(12):2277–93. 10.1089/ars.2007.1782 17979528

[65] Tu BP Weissman JS. Oxidative protein folding in eukaryotes: mechanisms and consequences. J Cell Biol (2004) 164(3):341–6. 10.1083/jcb.200311055 14757749 PMC2172237

[66] Malhotra JD Kaufman RJ The endoplasmic reticulum and the unfolded protein response. Semin Cell Dev Biol (2007) 18(6):716–31. 10.1016/j.semcdb.2007.09.003 18023214 PMC2706143

[67] Radermacher KA Wingler K Langhauser F Altenhofer S Kleikers P Hermans JJ Neuroprotection after stroke by targeting NOX4 as a source of oxidative stress. Antioxid Redox Signal (2013) 18(12):1418–27. 10.1089/ars.2012.4797 22937798 PMC3603500

[68] Bhandary B Marahatta A Kim HR Chae HJ. An involvement of oxidative stress in endoplasmic reticulum stress and its associated diseases. Int J Mol Sci (2012) 14(1):434–56. 10.3390/ijms14010434 23263672 PMC3565273

[69] Bylund J Brown KL Movitz C Dahlgren C Karlsson A. Intracellular generation of superoxide by the phagocyte NADPH oxidase: how, where, and what for? Free Radic Biol Med (2010) 49(12):1834–45. 10.1016/j.freeradbiomed.2010.09.016 20870019

[70] Kareyeva AV Grivennikova VG Vinogradov AD. Mitochondrial hydrogen peroxide production as determined by the pyridine nucleotide pool and its redox state. Biochim Biophys Acta (2012) 1817(10):1879–85. 10.1016/j.bbabio.2012.03.033 22503830

[71] Sosa V Moline T Somoza R Paciucci R Kondoh H ME LL. Oxidative stress and cancer: an overview. Ageing Res Rev (2013) 12(1):376–90. 10.1016/j.arr.2012.10.004 23123177

[72] Davies KJ. Oxidative stress: the paradox of aerobic life. Biochem Soc Symp (1995) 61:1–31. 10.1042/bss0610001 8660387

[73] Hildeman DA. Regulation of T-cell apoptosis by reactive oxygen species. Free Radic Biol Med (2004) 36(12):1496–504. 10.1016/j.freeradbiomed.2004.03.023 15182852

[74] Kalinina EV Chernov NN Saprin AN. Involvement of thio-, peroxi-, and glutaredoxins in cellular redox-dependent processes. Biochemistry (Mosc) (2008) 73(13):1493–510. 10.1134/S0006297908130099 19216714

[75] Schafer FQ Buettner GR. Redox environment of the cell as viewed through the redox state of the glutathione disulfide/glutathione couple. Free Radic Biol Med (2001) 30(11):1191–212. 10.1016/S0891-5849(01)00480-4 11368918

[76] Jones DP Redox potential of GSH/GSSG couple: assay and biological significance. Methods Enzymol (2002) 348:93–112. 10.1016/S0076-6879(02)48630-2 11885298

[77] Gonenc A Tokgoz D Aslan S Torun M. Oxidative stress in relation to lipid profiles in different stages of breast cancer. Indian J Biochem Biophys (2005) 42(3):190–4. 23923564

[78] Veskoukis AS Kyparos A Nikolaidis MG Stagos D Aligiannis N Halabalaki M The antioxidant effects of a polyphenol-rich grape pomace extract in vitro do not correspond in vivo using exercise as an oxidant stimulus. Oxid Med Cell Longev (2012) 2012:185867. 10.1155/2012/185867 22693650 PMC3368594

[79] Matsui A Ikeda T Enomoto K Hosoda K Nakashima H Omae K Increased formation of oxidative DNA damage, 8-hydroxy-2’-deoxyguanosine, in human breast cancer tissue and its relationship to GSTP1 and COMT genotypes. Cancer Lett (2000) 151(1):87–95. 10.1016/S0304-3835(99)00424-3 10766427

[80] D’Autreaux B Toledano MB. ROS as signalling molecules: mechanisms that generate specificity in ROS homeostasis. Nat Rev Mol Cell Biol (2007) 8(10):813–24. 10.1038/nrm2256 17848967

[81] Genestra M. Oxyl radicals, redox-sensitive signalling cascades and antioxidants. Cell Signal (2007) 19(9):1807–19. 10.1016/j.cellsig.2007.04.009 17570640

[82] Halliwell B. Free radicals and antioxidants – quo vadis? Trends Pharmacol Sci (2011) 32(3):125–30. 10.1016/j.tips.2010.12.002 21216018

[83] Gutowski M Kowalczyk S. A study of free radical chemistry: their role and pathophysiological significance. Acta Biochim Pol (2013) 60(1):1–16. 23513192

[84] Rubattu S Mennuni S Testa M Mennuni M Pierelli G Pagliaro B Pathogenesis of chronic cardiorenal syndrome: is there a role for oxidative stress? Int J Mol Sci (2013) 14(11):23011–32. 10.3390/ijms141123011 24264044 PMC3856103

[85] Arnold RS Shi J Murad E Whalen AM Sun CQ Polavarapu R Hydrogen peroxide mediates the cell growth and transformation caused by the mitogenic oxidase Nox1. Proc Natl Acad Sci U S A (2001) 98(10):5550–5. 11331784 10.1073/pnas.101505898 PMC33250

[86] Tojo T Ushio-Fukai M Yamaoka-Tojo M Ikeda S Patrushev N Alexander RW Role of gp91phox(Nox2)-containing NAD(P)H oxidase in angiogenesis in response to hindlimb ischemia. Circulation (2005) 111(18):2347–55. 15867174 10.1161/01.CIR.0000164261.62586.14

[87] Maciag A Sithanandam G Anderson LM. Mutant K-rasV12 increases COX-2, peroxides and DNA damage in lung cells. Carcinogenesis (2004) 25(11):2231–7. 10.1093/carcin/bgh245 15284181

[88] Weyemi U Dupuy C. The emerging role of ROS-generating NADPH oxidase NOX4 in DNA-damage responses. Mutat Res (2012) 751(2):77–81. 10.1016/j.mrrev.2012.04.002 22580379

[89] Altieri F Grillo C Maceroni M Chichiarelli S. DNA damage and repair: from molecular mechanisms to health implications. Antioxid Redox Signal (2008) 10(5):891–937. 10.1089/ars.2007.1830 18205545

[90] Cadet J Douki T Ravanat JL. Oxidatively generated base damage to cellular DNA. Free Radic Biol Med (2010) 49(1):9–21. 10.1016/j.freeradbiomed.2010.03.025 20363317

[91] De Bont R van Larebeke N. Endogenous DNA damage in humans: a review of quantitative data. Mutagenesis (2004) 19(3):169–85. 10.1093/mutage/geh025 15123782

[92] Sedelnikova OA Redon CE Dickey JS Nakamura AJ Georgakilas AG Bonner WM. Role of oxidatively induced DNA lesions in human pathogenesis. Mutat Res (2010) 704(1–3):152–9. 10.1016/j.mrrev.2009.12.005 20060490 PMC3074954

[93] Epe B. Role of endogenous oxidative DNA damage in carcinogenesis: what can we learn from repair-deficient mice? Biol Chem (2002) 383(3–4):467–75. 10.1515/BC.2002.049 12033436

[94] Jackson AL Loeb LA. The contribution of endogenous sources of DNA damage to the multiple mutations in cancer. Mutat Res (2001) 477(1–2):7–21. 10.1016/S0027-5107(01)00091-4 11376682

[95] Pollycove M Feinendegen LE. Radiation-induced versus endogenous DNA damage: possible effect of inducible protective responses in mitigating endogenous damage. Hum Exp Toxicol (2003) 22(6):290–306. 10.1191/0960327103ht370oa 12856953

[96] Georgakilas AG Mosley WG Georgakila S Ziech D Panayiotidis MI. Viral-induced human carcinogenesis: an oxidative stress perspective. Mol Biosyst (2010) 6(7):1162–72. 10.1039/b923958h 20436967

[97] Lim CH Dedon PC Deen WM. Kinetic analysis of intracellular concentrations of reactive nitrogen species. Chem Res Toxicol (2008) 21(11):2134–47. 10.1021/tx800213b 18828639 PMC3722615

[98] Sokolov MV Dickey JS Bonner WM Sedelnikova OA. gamma-H2AX in bystander cells: not just a radiation-triggered event, a cellular response to stress mediated by intercellular communication. Cell Cycle (2007) 6(18):2210–2. 10.4161/cc.6.18.4682 17881892

[99] Cohen G Sinet PM The Fenton reaction between ferrous-diethylenetriaminepentaacetic acid and hydrogen peroxide. FEBS Lett (1982) 138:258–60. 10.1016/0014-5793(82)80455-9

[100] Spassky A Angelov D. Influence of the local helical conformation on the guanine modifications generated from one-electron DNA oxidation. Biochemistry (1997) 36(22):6571–6. 10.1021/bi962761d 9184136

[101] Chen TH Chang PC Chang MC Lin YF Lee HM. Chloroquine induces the expression of inducible nitric oxide synthase in C6 glioma cells. Pharmacol Res (2005) 51(4):329–36. 10.1016/j.phrs.2004.10.004 15683746

[102] Vessoni AT Quinet A Andrade-Lima LC Martins DJ Garcia CC Rocha CR Chloroquine-induced glioma cells death is associated with mitochondrial membrane potential loss, but not oxidative stress. Free Radic Biol Med (2016) 90:91–100. 10.1016/j.freeradbiomed.2015.11.008 26577174

[103] Park BC Park SH Paek SH Park SY Kwak MK Choi HG Chloroquine-induced nitric oxide increase and cell death is dependent on cellular GSH depletion in A172 human glioblastoma cells. Toxicol Lett (2008) 178(1):52–60. 10.1016/j.toxlet.2008.02.003 18359172

[104] Lin CH Lin YF Chang MC Wu CH Ho YS Lee HM. Advanced glycosylation end products induce nitric oxide synthase expression in C6 glioma cells: involvement of a p38 MAP kinase-dependent mechanism. Life Sci (2001) 69(21):2503–15. 10.1016/S0024-3205(01)01330-3 11693258

[105] Gitika B Sai Ram M Sharma SK Ilavazhagan G Banerjee PK. Quercetin protects C6 glial cells from oxidative stress induced by tertiary-butylhydroperoxide. Free Radic Res (2006) 40(1):95–102. 10.1080/10715760500335447 16298764

[106] Panickar KS Anderson RA. Mechanisms underlying the protective effects of myricetin and quercetin following oxygen-glucose deprivation-induced cell swelling and the reduction in glutamate uptake in glial cells. Neuroscience (2011) 183:1–14. 10.1016/j.neuroscience.2011.03.064 21496478

[107] Vidak M Rozman D Komel R. Effects of flavonoids from food and dietary supplements on glial and glioblastoma multiforme cells. Molecules (2015) 20(10):19406–32. 10.3390/molecules201019406 26512639 PMC6332278

[108] Jakubowicz-Gil J Langner E Badziul D Wertel I Rzeski W. Apoptosis induction in human glioblastoma multiforme T98G cells upon temozolomide and quercetin treatment. Tumour Biol (2013) 34(4):2367–78. 10.1007/s13277-013-0785-0 23580181 PMC3713258

[109] Chen TJ Jeng JY Lin CW Wu CY Chen YC. Quercetin inhibition of ROS-dependent and -independent apoptosis in rat glioma C6 cells. Toxicology (2006) 223(1–2):113–26. 10.1016/j.tox.2006.03.007 16647178

[110] Agarwal A Sharma V Tewari R Koul N Joseph C Sen E. Epigallocatechin-3-gallate exhibits anti-tumor effect by perturbing redox homeostasis, modulating the release of pro-inflammatory mediators and decreasing the invasiveness of glioblastoma cells. Mol Med Rep (2008) 1(4):511–5. 10.3892/mmr.1.4.511 21479441

[111] Das A Banik NL Ray SK. Flavonoids activated caspases for apoptosis in human glioblastoma T98G and U87MG cells but not in human normal astrocytes. Cancer (2010) 116(1):164–76. 10.1002/cncr.24699 19894226 PMC3159962

[112] Chou YC Chang MY Wang MJ Liu HC Chang SJ Harnod T Phenethyl isothiocyanate alters the gene expression and the levels of protein associated with cell cycle regulation in human glioblastoma GBM 8401 cells. Environ Toxicol (2015). 10.1002/tox.22224 26678675

[113] Thida M Kim DW Tran TT Pham MQ Lee H Kim I Gambogic acid induces apoptotic cell death in T98G glioma cells. Bioorg Med Chem Lett (2015) 26(3):1097–101. 10.1016/j.bmcl.2015.11.043 26631318

[114] Kuete V Mbaveng AT Zeino M Fozing CD Ngameni B Kapche GD Cytotoxicity of three naturally occurring flavonoid derived compounds (artocarpesin, cycloartocarpesin and isobavachalcone) towards multi-factorial drug-resistant cancer cells. Phytomedicine (2015) 22(12):1096–102. 10.1016/j.phymed.2015.07.006 26547532

[115] Yuan S Lu Y Yang J Chen G Kim S Feng L Metabolic activation of mitochondria in glioma stem cells promotes cancer development through a reactive oxygen species-mediated mechanism. Stem Cell Res Ther (2015) 6:198. 10.1186/s13287-015-0174-2 26472041 PMC4606508

[116] Aroui S Dardevet L Ajmia WB de Boisvilliers M Perrin F Laajimi A A novel platinum-maurocalcine conjugate induces apoptosis of human glioblastoma cells by acting through the ROS-ERK/AKT-p53 pathway. Mol Pharm (2015) 12(12):4336–48. 10.1021/acs.molpharmaceut.5b00531 26465677

[117] Shi L Sun G. Low-dose DMC significantly enhances the effect of TMZ on glioma cells by targeting multiple signaling pathways both in vivo and in vitro. Neuromolecular Med (2015) 17(4):431–42. 10.1007/s12017-015-8372-8 26458914

[118] Di Bari M Tombolillo V Conte C Castigli E Sciaccaluga M Iorio E Cytotoxic and genotoxic effects mediated by M2 muscarinic receptor activation in human glioblastoma cells. Neurochem Int (2015) 90:261–70. 10.1016/j.neuint.2015.09.008 26455407

[119] Singer E Judkins J Salomonis N Matlaf L Soteropoulos P McAllister S Reactive oxygen species-mediated therapeutic response and resistance in glioblastoma. Cell Death Dis (2015) 6:e1601. 10.1038/cddis.2014.566 25590811 PMC4669764

[120] Zhang FJ Yang JY Mou YH Sun BS Wang JM Wu CF Oligomer procyanidins from grape seeds induce a paraptosis-like programmed cell death in human glioblastoma U-87 cells. Pharm Biol (2010) 48:883–90. 10.3109/13880200903311102 20673175

[121] Khan M Yi F Rasul A Li T Wang N Gao H Alantolactone induces apoptosis in glioblastoma cells via GSH depletion, ROS generation, and mitochondrial dysfunction. IUBMB Life (2012) 64(9):783–94. 10.1002/iub.1068 22837216

[122] Macchioni L Davidescu M Sciaccaluga M Marchetti C Migliorati G Coaccioli S Mitochondrial dysfunction and effect of antiglycolytic bromopyruvic acid in GL15 glioblastoma cells. J Bioenerg Biomembr (2011) 43(5):507–18. 10.1007/s10863-011-9375-2 21833601

[123] Keir ST Dewhirst MW Kirkpatrick JP Bigner DD Batinic-Haberle I. Cellular redox modulator, ortho Mn(III) meso-tetrakis(N-n-hexylpyridinium-2-yl)porphyrin, MnTnHex-2-PyP(5+) in the treatment of brain tumors. Anticancer Agents Med Chem (2011) 11(2):202–12. 10.2174/187152011795255957 21291403 PMC3357315

[124] Sharma V Joseph C Ghosh S Agarwal A Mishra MK Sen E. Kaempferol induces apoptosis in glioblastoma cells through oxidative stress. Mol Cancer Ther (2007) 6(9):2544–53. 10.1158/1535-7163.MCT-06-0788 17876051

[125] Shen H Decollogne S Dilda PJ Hau E Chung SA Luk PP Dual-targeting of aberrant glucose metabolism in glioblastoma. J Exp Clin Cancer Res (2015) 34:14. 10.1186/s13046-015-0130-0 25652202 PMC4324653

[126] Michelakis ED Sutendra G Dromparis P Webster L Haromy A Niven E Metabolic modulation of glioblastoma with dichloroacetate. Sci Transl Med (2010) 2(31):31ra34. 10.1126/scitranslmed.3000677 20463368

[127] Klingelhoeffer C Kammerer U Koospal M Muhling B Schneider M Kapp M Natural resistance to ascorbic acid induced oxidative stress is mainly mediated by catalase activity in human cancer cells and catalase-silencing sensitizes to oxidative stress. BMC Complement Altern Med (2012) 12:61. 10.1186/1472-6882-12-61 22551313 PMC3404974

[128] Festa M Capasso A D’Acunto CW Masullo M Rossi AG Pizza C Xanthohumol induces apoptosis in human malignant glioblastoma cells by increasing reactive oxygen species and activating MAPK pathways. J Nat Prod (2011) 74(12):2505–13. 10.1021/np200390x 22111577

[129] Eom KS Kim HJ So HS Park R Kim TY. Berberine-induced apoptosis in human glioblastoma T98G cells is mediated by endoplasmic reticulum stress accompanying reactive oxygen species and mitochondrial dysfunction. Biol Pharm Bull (2010) 33(10):1644–9. 10.1248/bpb.33.1644 20930370

[130] Iida M Doi H Asamoto S Sugiyama H Sakagami H Kuribayashi N Effect of glutathione-modulating compounds on platinum compounds-induced cytotoxicity in human glioma cell lines. Anticancer Res (1999) 19(6B):5383–4. 10697565

[131] Iida M Sunaga S Hirota N Kuribayashi N Sakagami H Takeda M Effect of glutathione-modulating compounds on hydrogen-peroxide-induced cytotoxicity in human glioblastoma and glioma cell lines. J Cancer Res Clin Oncol (1997) 123(11–12):619–22. 10.1007/s004320050115 9620220 PMC12201900

[132] Babior BM NADPH oxidase: an update. Blood (1999) 93(5):1464–76. 10029572

[133] Chiarugi P Fiaschi T. Redox signalling in anchorage-dependent cell growth. Cell Signal (2007) 19(4):672–82. 10.1016/j.cellsig.2006.11.009 17204396

[134] Storz P. Reactive oxygen species in tumor progression. Front Biosci (2005) 10:1881–96. 10.2741/1667 15769673

[135] Scherz-Shouval R Elazar Z. ROS, mitochondria and the regulation of autophagy. Trends Cell Biol (2007) 17(9):422–7. 10.1016/j.tcb.2007.07.009 17804237

[136] Baas AS Berk BC. Differential activation of mitogen-activated protein kinases by H_2_O_2_ and O_2_^−^ in vascular smooth muscle cells. Circ Res (1995) 77(1):29–36. 10.1161/01.RES.77.1.29 7540516

[137] Devary Y Gottlieb RA Smeal T Karin M. The mammalian ultraviolet response is triggered by activation of Src tyrosine kinases. Cell (1992) 71(7):1081–91. 10.1016/S0092-8674(05)80058-3 1473146

[138] Khandrika L Kumar B Koul S Maroni P Koul HK Oxidative stress in prostate cancer. Cancer Lett (2009) 282(2):125–36. 10.1016/j.canlet.2008.12.011 19185987 PMC2789743

[139] Martinez-Pastor B Mostoslavsky R. Sirtuins, metabolism, and cancer. Front Pharmacol (2012) 3:22. 10.3389/fphar.2012.00022 22363287 PMC3282920

[140] Kassenbrock CK Hunter S Garl P Johnson GL Anderson SM. Inhibition of Src family kinases blocks epidermal growth factor (EGF)-induced activation of Akt, phosphorylation of c-Cbl, and ubiquitination of the EGF receptor. J Biol Chem (2002) 277(28):24967–75. 10.1074/jbc.M201026200 11994282

[141] Li Z Dong T Proschel C Noble M. Chemically diverse toxicants converge on Fyn and c-Cbl to disrupt precursor cell function. PLoS Biol (2007) 5(2):e35. 10.1371/journal.pbio.0050035 17298174 PMC1790953

[142] Huang PH Xu AM White FM. Oncogenic EGFR signaling networks in glioma. Sci Signal (2009) 2(87):re6. 10.1126/scisignal.287re6 19738203

[143] Normanno N De Luca A Bianco C Strizzi L Mancino M Maiello MR Epidermal growth factor receptor (EGFR) signaling in cancer. Gene (2006) 366(1):2–16. 10.1016/j.gene.2005.10.018 16377102

[144] Stevens BM Folts CJ Cui W Bardin AL Walter K Carson-Walter E Cool-1-mediated inhibition of c-Cbl modulates multiple critical properties of glioblastomas, including the ability to generate tumors in vivo. Stem Cells (2014) 32(5):1124–35. 10.1002/stem.1644 24458840

[145] Fukami A Adachi H Yamagishi S Matsui T Ueda S Nakamura K Factors associated with serum high mobility group box 1 (HMGB1) levels in a general population. Metabolism (2009) 58(12):1688–93. 10.1016/j.metabol.2009.05.024 19616266

[146] Andersson U Tracey KJ. HMGB1 is a therapeutic target for sterile inflammation and infection. Annu Rev Immunol (2011) 29:139–62. 10.1146/annurev-immunol-030409-101323 21219181 PMC4536551

[147] Wang H Bloom O Zhang M Vishnubhakat JM Ombrellino M Che J HMG-1 as a late mediator of endotoxin lethality in mice. Science (1999) 285(5425):248–51. 10.1126/science.285.5425.248 10398600

[148] Kang R Zhang Q Zeh HJ III Lotze MT Tang D. HMGB1 in cancer: good, bad, or both? Clin Cancer Res (2013) 19(15):4046–57. 10.1158/1078-0432.CCR-13-0495 23723299 PMC3732559

[149] Stros M. HMGB proteins: interactions with DNA and chromatin. Biochim Biophys Acta (2010) 1799(1–2):101–13. 10.1016/j.bbagrm.2009.09.008 20123072

[150] Tang D Kang R Livesey KM Zeh HJ III Lotze MT. High mobility group box 1 (HMGB1) activates an autophagic response to oxidative stress. Antioxid Redox Signal (2011) 15(8):2185–95. 10.1089/ars.2010.3666 21395369 PMC3166205

[151] Galdiero MR Bonavita E Barajon I Garlanda C Mantovani A Jaillon S. Tumor associated macrophages and neutrophils in cancer. Immunobiology (2013) 218(11):1402–10. 10.1016/j.imbio.2013.06.003 23891329

[152] Cao Z Shang B Zhang G Miele L Sarkar FH Wang Z Tumor cell-mediated neovascularization and lymphangiogenesis contrive tumor progression and cancer metastasis. Biochim Biophys Acta (2013) 1836(2):273–86. 10.1016/j.bbcan.2013.08.001 23933263

[153] Qian BZ Pollard JW. Macrophage diversity enhances tumor progression and metastasis. Cell (2010) 141(1):39–51. 10.1016/j.cell.2010.03.014 20371344 PMC4994190

[154] Ruffell B Affara NI Coussens LM. Differential macrophage programming in the tumor microenvironment. Trends Immunol (2012) 33(3):119–26. 10.1016/j.it.2011.12.001 22277903 PMC3294003

[155] Kovacic P Wakelin LP. Review: DNA molecular electrostatic potential: novel perspectives for the mechanism of action of anticancer drugs involving electron transfer and oxidative stress. Anticancer Drug Des (2001) 16(4–5):175–84. 12049476

[156] Schumacker PT. Reactive oxygen species in cancer cells: live by the sword, die by the sword. Cancer Cell (2006) 10(3):175–6. 10.1016/j.ccr.2006.08.015 16959608

[157] Cobbs CS Whisenhunt TR Wesemann DR Harkins LE Van Meir EG Samanta M. Inactivation of wild-type p53 protein function by reactive oxygen and nitrogen species in malignant glioma cells. Cancer Res (2003) 63(24):8670–3. 14695179

[158] Tennant AH Kligerman AD. Superoxide dismutase protects cells from DNA damage induced by trivalent methylated arsenicals. Environ Mol Mutagen (2011) 52(3):238–43. 10.1002/em.20609 20740636

[159] Warburg O On the origin of cancer cells. Science (1956) 123(3191):309–14. 13298683 10.1126/science.123.3191.309

[160] Bartoletti-Stella A Mariani E Kurelac I Maresca A Caratozzolo MF Iommarini L Gamma rays induce a p53-independent mitochondrial biogenesis that is counter-regulated by HIF1alpha. Cell Death Dis (2013) 4:e663 10.1038/cddis.2013.187 23764844 PMC3702280

[161] de Groof AJ te Lindert MM van Dommelen MM Wu M Willemse M Smift AL Increased OXPHOS activity precedes rise in glycolytic rate in H-RasV12/E1A transformed fibroblasts that develop a Warburg phenotype. Mol Cancer (2009) 8:54. 10.1186/1476-4598-8-54 19646236 PMC2734543

[162] El Mjiyad N Caro-Maldonado A Ramirez-Peinado S Munoz-Pinedo C. Sugar-free approaches to cancer cell killing. Oncogene (2011) 30(3):253–64. 10.1038/onc.2010.466 20972457

[163] Ramsay EE Hogg PJ Dilda PJ. Mitochondrial metabolism inhibitors for cancer therapy. Pharm Res (2011) 28(11):2731–44. 10.1007/s11095-011-0584-5 21918915

[164] Abildgaard C Dahl C Basse AL Ma T Guldberg P. Bioenergetic modulation with dichloroacetate reduces the growth of melanoma cells and potentiates their response to BRAFV600E inhibition. J Transl Med (2014) 12:247. 10.1186/s12967-014-0247-5 25182332 PMC4156963

[165] Ruggieri S Orsomando G Sorci L Raffaelli N. Regulation of NAD biosynthetic enzymes modulates NAD-sensing processes to shape mammalian cell physiology under varying biological cues. Biochim Biophys Acta (2015) 1854(9):1138–49. 10.1016/j.bbapap.2015.02.021 25770681

[166] Easton JB Houghton PJ mTOR and cancer therapy. Oncogene (2006) 25(48):6436–46. 10.1038/sj.onc.1209886 17041628

[167] Sun Q Chen X Ma J Peng H Wang F Zha X Mammalian target of rapamycin up-regulation of pyruvate kinase isoenzyme type M2 is critical for aerobic glycolysis and tumor growth. Proc Natl Acad Sci U S A (2011) 108(10):4129–34. 10.1073/pnas.1014769108 21325052 PMC3054028

[168] Wittwer JA Robbins D Wang F Codarin S Shen X Kevil CG Enhancing mitochondrial respiration suppresses tumor promoter TPA-induced PKM2 expression and cell transformation in skin epidermal JB6 cells. Cancer Prev Res (Phila) (2011) 4(9):1476–84. 10.1158/1940-6207.CAPR-11-0028 21673231 PMC4827450

[169] Ferretti M Fabbiano C Di Bari M Ponti D Calogero A Tata AM. M2 muscarinic receptors inhibit cell proliferation in human glioblastoma cell lines. Life Sci (2012) 91(21–22):1134–7. 10.1016/j.lfs.2012.04.033 22575825

[170] Kim H Moon JY Ahn KS Cho SK. Quercetin induces mitochondrial mediated apoptosis and protective autophagy in human glioblastoma U373MG cells. Oxid Med Cell Longev (2013) 2013:596496. 10.1155/2013/596496 24379902 PMC3863523

[171] Badziul D Jakubowicz-Gil J Langner E Rzeski W Glowniak K Gawron A. The effect of quercetin and imperatorin on programmed cell death induction in T98G cells in vitro. Pharmacol Rep (2014) 66(2):292–300. 10.1016/j.pharep.2013.10.003 24911084

[172] Jakubowicz-Gil J Langner E Badziul D Wertel I Rzeski W. Quercetin and sorafenib as a novel and effective couple in programmed cell death induction in human gliomas. Neurotox Res (2014) 26(1):64–77. 10.1007/s12640-013-9452-x 24366851 PMC4035551

[173] Zhang Y Liu Q Wang F Ling EA Liu S Wang L Melatonin antagonizes hypoxia-mediated glioblastoma cell migration and invasion via inhibition of HIF-1alpha. J Pineal Res (2013) 55(2):121–30. 10.1111/jpi.12052 23551342

[174] Kohsaka S Takahashi K Wang L Tanino M Kimura T Nishihara H Inhibition of GSH synthesis potentiates temozolomide-induced bystander effect in glioblastoma. Cancer Lett (2013) 331(1):68–75. 10.1016/j.canlet.2012.12.005 23246370

[175] Anderson CP Matthay KK Perentesis JP Neglia JP Bailey HH Villablanca JG Pilot study of intravenous melphalan combined with continuous infusion L-S,R-buthionine sulfoximine for children with recurrent neuroblastoma. Pediatr Blood Cancer (2015) 62(10):1739–46. 10.1002/pbc.25594 26153194

[176] Dukhande VV Kawikova I Bothwell AL Lai JC. Neuroprotection against neuroblastoma cell death induced by depletion of mitochondrial glutathione. Apoptosis (2013) 18(6):702–12. 10.1007/s10495-013-0836-4 23494481 PMC3645366

[177] Li Q Yin X Wang W Zhan M Zhao B Hou Z The effects of buthionine sulfoximine on the proliferation and apoptosis of biliary tract cancer cells induced by cisplatin and gemcitabine. Oncol Lett (2016) 11(1):474–80. 10.3892/ol.2015.3879 26870236 PMC4727028

[178] Dringen R Hirrlinger J Glutathione pathways in the brain. Biol Chem (2003) 384(4):505–16. 10.1515/BC.2003.059 12751781

[179] Raps SP Lai JC Hertz L Cooper AJ. Glutathione is present in high concentrations in cultured astrocytes but not in cultured neurons. Brain Res (1989) 493(2):398–401. 10.1016/0006-8993(89)91178-5 2765907

[180] Herst PM Broadley KW Harper JL McConnell MJ. Pharmacological concentrations of ascorbate radiosensitize glioblastoma multiforme primary cells by increasing oxidative DNA damage and inhibiting G2/M arrest. Free Radic Biol Med (2012) 52(8):1486–93. 10.1016/j.freeradbiomed.2012.01.021 22342518

